# Comparing a Novel Malformation Syndrome Caused by Pathogenic Variants in *FBRSL1* to AUTS2 Syndrome

**DOI:** 10.3389/fcell.2021.779009

**Published:** 2021-11-05

**Authors:** Silke Pauli, Hanna Berger, Roser Ufartes, Annette Borchers

**Affiliations:** ^1^ Institute of Human Genetics, University Medical Center Göttingen, Göttingen, Germany; ^2^ Faculty of Biology, Molecular Embryology, Philipps‐University Marburg, Marburg, Germany

**Keywords:** FBRSL1, AUTS2, malformation syndrome, embryonic development, polycomb complex

## Abstract

Truncating variants in specific exons of *Fibrosin-like protein 1* (*FBRSL1*) were recently reported to cause a novel malformation and intellectual disability syndrome. The clinical spectrum includes microcephaly, facial dysmorphism, cleft palate, skin creases, skeletal anomalies and contractures, postnatal growth retardation, global developmental delay as well as respiratory problems, hearing impairment and heart defects. The function of FBRSL1 is largely unknown, but pathogenic variants in the *FBRSL1* paralog *Autism Susceptibility Candidate 2* (*AUTS2*) are causative for an intellectual disability syndrome with microcephaly (AUTS2 syndrome). Some patients with AUTS2 syndrome also show additional symptoms like heart defects and contractures overlapping with the phenotype presented by patients with *FBRSL1* mutations. For AUTS2, a dual function, depending on different isoforms, was described and suggested for FBRSL1. Both, nuclear FBRSL1 and AUTS2 are components of the Polycomb subcomplexes PRC1.3 and PRC1.5. These complexes have essential roles in developmental processes, cellular differentiation and proliferation by regulating gene expression via histone modification. In addition, cytoplasmic AUTS2 controls neural development, neuronal migration and neurite extension by regulating the cytoskeleton. Here, we review recent data on FBRSL1 in respect to previously published data on AUTS2 to gain further insights into its molecular function, its role in development as well as its impact on human genetics.

## FBRSL1 Variants Cause a Novel Disability Syndrome With an Overlapping Phenotype to AUTS2 Syndrome

Recently, we identified truncating variants in the *FBRSL1* gene in three unrelated children with an unknown malformation syndrome (Ufartes et al., 2020). The patients presented with respiratory insufficiency and feeding difficulties in the neonatal period. During infancy, intellectual disability, no active speech, postnatal microcephaly, growth retardation and contractures became apparent. In addition, two of the three patients showed cleft palate and heart defects (one with atrial septal defect and persistent ductus arteriosus, one with atrial septal defect and ventricular septal defect). In one patient asplenia and in another patient anal atresia were observed. Furthermore, the two more severely affected patients were born with pronounced congenital skin creases at the back, the arms, and legs. During the first year of life the skin creases became less pronounced and disappeared (Ufartes et al., 2020). Interestingly, the clinical phenotype of the newly described malformation syndrome caused by *FBRSL1* variants partially overlaps with the severe form of AUTS2 syndrome (Table 1).

**TABLE 1:** Comparison of clinical features seen in patients with *FBRSL1* mutation and patients with AUTS2 syndrome.

Clinical findings	FBRSL1 syndromic phenotype	AUTS2 syndrome
**Growth and feeding**		
Low birth weight	2/3	10/54 (18,5%)
Short stature	3/3	26/59 (44,1%)
Microcephaly	3/3	37/57 (64,9%)
Feeding difficulties	3/3	33/55 (60,0%)
**Neurodevelopmental disorders**		
Intellectual disability	3/3	64/66 (97,0%)
Autism/autistic behaviour	3/3	16/40 (40,0%)
Sound sensitivity	n.a	28/56 (50,0%)
Hyperactivity/ADHD	n.a	17/28 (60,7%)
**Neurological disorders**		
Generalized hypotonia	n.a	23/60 (38,3%)
Structural brain anomaly	-/1/n.a	11/46 (23,9%)
Cerebral palsy/spasticity	2/3	20/57 (35,1%)
Other: respiratory insufficiency with ventilation therapy	3/3	
**Dysmorphic features**		
High arched eyebrows	2/3	13/37 (35,1%)
Hypertelorism	0/3	14/37 (37,8%)
Proptosis	0/3	7/37 (18,9%)
Short palpebral fissures	0/3	9/37 (24,3%)
Up slanting palpebral fissures	0/3	5/37 (13,5%)
Ptosis	0/3	11/37 (29,7%)
Epicanthol folds	2/3	8/37 (21,6%)
Strabismus	0/3	9/37 (24,3%)
Prominent nasal tip	1/3	7/37 (18,9%)
Anteverted nares	0/3	7/37 (18,9%)
Deep/broad nasal bridge	2/3	12/37 (32,4%)
Short/upturned philtrum	2/3	11/37 (29,7%)
Micro-/retrognatia	1/3	11/36 (30,6%)
Low set ears	2/3	11/36 (30,6%)
Ear pit	0/3	5/36 (13,9%)
Narrow mouth	1/3	16/37 (43,2%)
Other: widely spaced teeth	2/3	-
**Skeletal disorders**		
Kyphosis/scoliosis	3/3	10/47 (21,3%)
Arthrogryposis/shallow palmar creases	0/3	6/28 (21,4%)
Tight heel cords	n.a	6/13 (46,2%)
Other (camptodactyly/contractures)	3/3	-
**Congenital malformation**		
Hernia umbilicalis/inguinalis	0/3	6/59 (10,2%)
Patent foramen ovale/atrial septum defect	2/3	4/26 (15,4%)
Other		
•Cleft palate	2/3	-
•Asplenia	1/3	-
•Anal atresia	1/3	-
•Abnormality of the skin	2/3	-
•Hearing impairment	2/3	-

The clinical feature terminology is based on the list of features used for the AUTS2 syndrome severity scoring system (Beunders et al., 2013). The data for AUTS2 syndrome were adapted from Sanchez-Jimeno et al. which is based on nine different studies (Sultana et al., 2002; Kalscheuer et al., 2007; Bakkaloglu et al., 2008; Huang et al., 2010; Girirajan et al., 2011; Jolley et al., 2013; Nagamani et al., 2013; Liu et al., 2015; Beunders et al., 2016). In addition, the data include a cohort of five patients published by Sanchez-Jimeno et al. (Sanchez-Jimeno et al., 2021). The data for the FBRSL1 syndromic phenotype is based on the three patients published in Ufartes et al. (Ufartes et al., 2020). Abbreviations: n.a. = not assessed, ADHD = attention deficit/hyperactivity disorder. A remarkable overlap between the two syndromes was observed with a wider spectrum and higher rate of congenital malformations in children with a pathogenic variant in FBRSL1.

AUTS2 syndrome (MIM 615834) was first described in 2013 (Beunders et al., 2013), as a neurodevelopmental disorder caused by pathogenic variants and deletions of the *AUTS2* gene (MIM 607270, activator of transcription and developmental regulator). Depending on the location of *AUTS2* point mutations/deletions the phenotype ranges from an isolated neurodevelopmental disorder (e.g., autism spectrum disorder, attention deficit hyperactivity disorder, learning disabilities and/or intellectual disability) to a syndromic disorder with microcephaly, short stature, feeding difficulties, heart defects, skeletal anomalies, contractures and dysmorphic features (Beunders et al., 2013; Beunders et al., 2016; Saeki et al., 2019; Sanchez-Jimeno et al., 2021). To date, more than 60 patients with AUTS2 syndrome have been described in the literature and most of them carry an intragenic *de novo* deletion of *AUTS2*, whereas point mutations leading to the disease are rarely described (Sanchez-Jimeno et al., 2021). Due to a high inter- and intrafamilial variability an AUTS2 syndrome severity scoring system (ASSS) was established by Beunders and colleagues 2013. The scoring system is based on 32 features seen with a frequency of over 10% in AUTS2 syndrome patients of the first described cohort (Beunders et al., 2013). The ASSS revealed that patients with small deletions at the N-terminus of *AUTS2* typically present a mild phenotype; in some cases, these deletions were inherited from a mildly or unaffected parent (Beunders et al., 2013). In contrast, deletions of the C-terminus of AUTS2 are mostly associated with a severe AUTS2 syndrome phenotype combining neurodevelopmental features with malformations and dysmorphic features (Beunders et al., 2013). Therefore, it was suggested that the AUTS2 C-terminus plays a critical role in AUTS2 syndrome (Beunders et al., 2013; Beunders et al., 2016; Saeki et al., 2019; Sanchez-Jimeno et al., 2021). Interestingly, the situation seems to be different for the truncating *FBRSL1* variants characterized in Ufartes et al. (2020), which all localized to the N-terminus of the *FBRLS1* gene. As the patients carrying *FBRSL1* variants showed features associated with AUTS2 syndrome, we also used the ASSS to compare the phenotype of the three patients with a variant in *FBRSL1* (Ufartes et al., 2020) to patients with AUTS2 syndrome (Table 1). A remarkable clinical overlap between the FBRSL1 syndromic phenotype and the severe form of AUTS2 syndrome was observed. Although, so far only three patients with the FBRSL1 syndromic phenotype have been described (Ufartes et al., 2020), it seems that they show a wider range of congenital malformations compared to the symptoms observed in AUTS2 patients. To gain insight into common and distinct functions of FBRSL1 and AUTS2, we take a closer look at their evolutionary conservation and potential functions.

## FBRSL1 and AUTS2 Are Paralogs That Likely Share Conserved Functions

FBRSL1 and AUTS2 belong to a tripartite gene family, the AUTS2 family, which also includes Fibrosin (FBRS) (Singh et al., 2015). The AUTS2 family is predicted to be an ohnolog gene family (Singh et al., 2015), representing a group of paralog genes generated by two rounds of whole genome duplication during vertebrate evolution and frequently implicated in human disease (Dickerson and Robertson, 2012; Singh et al., 2012; Malaguti et al., 2014; Mclysaght et al., 2014). The AUTS2 family ohnologs show a large overlap of conserved regions, but also unique elements which likely contribute to the functional diversity of the proteins (Sellers et al., 2020). Detailed information about the conserved regions shared by AUTS2-related proteins as well as an evolutionary analysis of the AUTS2 family can be found in Sellers et al., 2020 (Sellers et al., 2020). Based on their extended phylogenetic analysis, Sellers et al. recommended to rename FBRSL1 to AUTS2-like Protein 1, because AUTS2 and FBRSL1 share a most recent common ancestor, suggesting that these proteins are evolutionary closer related to each other than to FBRS (Sellers et al., 2020). Thus, it is intriguing to speculate that both proteins may share common functions, which may also explain their overlapping phenotypes observed in the respective syndromes.

Research using animal model systems indicate that FBRSL1 and AUTS2 share common functions in vertebrate development. As Auts2 function in neurodevelopmental disorders has been addressed in a number of comprehensive reviews (Oksenberg and Ahituv, 2013; Hori and Hoshino, 2017; Pang et al., 2021), we will here only briefly discuss its role in mouse and zebrafish development. In the mouse, *Auts2* is broadly expressed in the developing brain, with high expression in key areas of higher cognitive brain function (Bedogni et al., 2010). Heterozygous disruption of *Auts2* results in similar symptoms as seen in AUTS2 syndrome patients including growth reduction, defects in communication, exploratory behavior as well as learning and memory, while social behavior and sensor motor gating functions were normal (Gao et al., 2014; Hori et al., 2015). In zebrafish, *auts2* is highly expressed in the developing brain and Morpholino-mediated knockdown resulted in microcephaly, reduced lower jaws, swimming defects and a reduced response to tactile stimuli (Beunders et al., 2013; Oksenberg et al., 2013).

**FIGURE 1:**
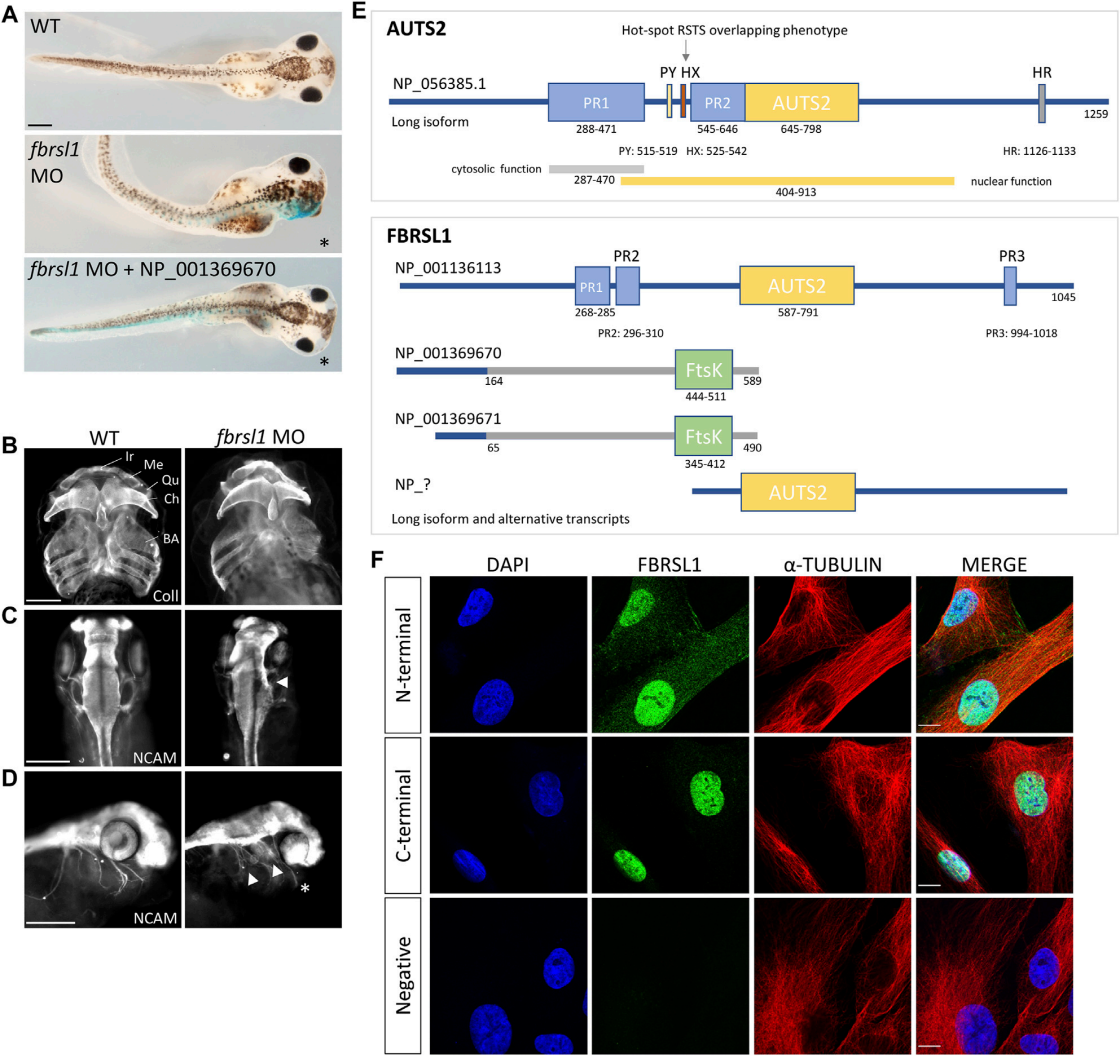
Fbrsl1 knockdown phenotypes in *Xenopus laevis* and cellular localization of distinct human *FBRSL1* transcripts. **(A)** Knockdown of Fbrsl1 by injection of a *fbrsl1* Morpholino oligonucleotide (MO) leads to craniofacial defects that can be rescued by co-injection of RNA coding for the human short N-terminal *FBRSL1 isoform NP_001369670* (Ufartes et al., 2020). *LacZ RNA* was co-injected as a lineage tracer, the injected side is marked by blue staining. **(B)** Anti-Collagen Type II immunofluorescence visualizes the cartilage and indicates cartilage hypoplasia on the *fbrsl1* MO-injected side. BA, branchial arches; Ch, ceratohyal; Ir, infrarostral; Me, Meckel’s cartilage; Qu, quadrate. **(C)** Neural cell adhesion molecule (NCAM) staining shows reduced brain size and **(D)** impaired outgrowth of cranial nerves on the *fbrsl1* MO-injected side; * marks the injected side. Scale bar in A-D: 500 µm. **(E)** Human *FBRSL1* transcripts/isoforms compared to the human AUTS2 long isoform as previously published (Sultana et al., 2002; Oksenberg and Ahituv, 2013; Sellers et al., 2020). Like its AUTS2 ohnolog, the long isoform has a AUTS2 domain and proline rich (PR) regions (predicted with MobiDB (Piovesan et al., 2020)). Both short N-terminal isoforms differ in their C-terminal sequence from the long isoform (presented in grey) due to an alternative exon 3, which contains an Ftsk-domain. In addition, a predicted C-terminal isoform (marked with “?“) including the AUTS2 domain is shown as this isoform was validated for mouse Fbrsl1. PR, proline-rich domain; PY, PPPY motif; HX, hexanucleotide repeat; HR, trinucleotide (H) repeat; AUTS2, AUTS2/FBRSL1/FBRSL homology region. **(F)** Immunofluorescence analysis performed on human fibroblasts. Antibodies directed against the N-terminal as well as the C-terminal part of FBRSL1 detected FBRSL1 isoforms (green) in the nucleus. However, only the N-terminal FBRSL1 antibody also detected FBRSL1 in the cytoplasm, suggesting that the short N-terminal FBRSL1 isoforms show cytoplasmic and nuclear localization. The negative control showed no signal. Cytoskeletal staining was detected using an *α*-Tubulin antibody and nuclei were stained using DAPI. Images were obtained using a confocal laser microscope with ×600 magnification. Scale Bar: 10 µm. All experimental data have been previously published (Ufartes et al., 2020).

Currently, data analyzing the function of Fbrsl1 in animal model systems are limited. The expression of *fbrsl1* has been analyzed in zebrafish and these data show that it is mainly expressed in the developing brain, but also in the spinal cord, the cranial ganglia and the somites (Kondrychyn et al., 2017). In *Xenopus*, *fbrsl1* is expressed throughout early developmental stages (Ufartes et al., 2020). At tailbud stages, it is expressed in the brain and craniofacial structures including the branchial arches and the cranial nerves (Ufartes et al., 2020). Morpholino-mediated Fbrsl1 knockdown resulted in craniofacial defects and the embryos showed cartilage hypoplasia as well as a reduction in brain size on the injected side (Figures 1A–C). Furthermore, the cranial nerves (Figure 1D) and motor neurons displayed impaired neuronal migration (Ufartes et al., 2020). Thus, the first functional data on Fbrsl1 in *Xenopus* development indicate that FBRSL1 may share similar functions with AUTS2 in neural development, but may also have a unique role in craniofacial development, which is also consistent with the findings in patients affected by the respective disorders.

## AUTS2 has Nuclear and Cytoplasmic Functions Which May Be Shared by FBRSL1

For AUTS2 a dual function, acting either in the cytoplasm or in the nucleus of developing neurons has been described (Hori et al., 2014). In the nucleus, AUTS2 was identified as a component of the Polycomb repressive complex PRC1 (Gao et al., 2012; Gao et al., 2014). Polycomb repressive complexes are multiprotein complexes acting as epigenetic regulators during development (Aranda et al., 2015; Chittock et al., 2017). Traditionally, they exert their function as transcriptional repressors (Simon and Kingston, 2013; Chittock et al., 2017; Kassis et al., 2017). The two main Polycomb complexes are PRC1 and PRC2 (reviewed in Barbour et al., 2020; Cohen et al., 2020; Geng and Gao, 2020). The PRC1 complex acts as an E3 ubiquitin ligase that monoubiquitinates lysine 119 of histone H2A (H2AK119ub1) (De Napoles et al., 2004; Wang et al., 2004). The consequences of Polycomb dependent histone H2A ubiquitination were recently reviewed by Tamburri et al. (2021). In addition, the PRC1 complex is involved in ubiquitination-independent chromatin compaction (Eskeland et al., 2010). At least six PRC1 subcomplexes (PRC1.1-PRC1.6) were identified consisting of the E3 ubiquitin ligase RING1A or RING1B and one of the six Polycomb Group Ring Fingers (PCGF1-6) (Chittock et al., 2017; Varlet et al., 2020). AUTS2 has been described as a component of the subcomplexes PRC1.3 and PRC1.5 (Gao et al., 2012; Gao et al., 2014). The PRC1.5 complex contains the components AUTS2, PCGF5, RING1B, CK2B, and RYBP (Gao et al., 2012; Gao et al., 2014). The binding of AUTS2 to the PRC1.5 complex switches its function to a transcriptional activator by recruiting the histone acetyltransferase EP300 and casein kinase 2 (CK2) (Gao et al., 2014; Liu et al., 2021). Co-immunoprecipitation experiments revealed that RING1B interacts with AUTS2 only in the presence of PCGF5 (Gao et al., 2014), suggesting a bridging function of PCGF5. The recruitment of CK2 to the complex is likely mediated by direct AUTS2 binding and this interaction suppresses monoubiquitination of H2AK119 by RING1B (Gao et al., 2014). The C-terminal part of AUTS2 (404–913) is sufficient to mediate the transcriptional activation via EP300 binding (Gao et al., 2014). Therefore, the recruitment of CK2 to the PRC1.5 complex and the AUTS2-EP300 interaction seem to be responsible for converting the repressive PRC1 function into an activator function (Gao et al., 2014; Monderer-Rothkoff et al., 2021). Recently, *de novo* pathogenic variants in the HX repeat region *of AUTS2* were described in patients with a phenotype overlapping with Rubinstein-Taybi syndrome (Liu et al., 2021). Rubinstein-Taybi syndrome (RSTS, OMIM 180849 and OMIM 613684) is a neurodevelopmental disorder characterized by intellectual disability, autism spectrum disorders, microcephaly, facial dysmorphism, growth retardation, large thumbs and hallux and a variable degree of additional malformations and symptoms (reviewed in Van Gils et al., 2021). The underlying cause of RSTS are pathogenic variants in *EP300* and *CREBBP* (Petrij et al., 1995; Roelfsema et al., 2005). Interestingly, the *AUTS2* variants leading to an RSTS-overlapping phenotype disrupt the binding of AUTS2 to EP300, suggesting that the HX repeat domain is responsible for this interaction (Liu et al., 2021). The binding of AUTS2 to PRC1.3 and the recruitment to chromatin was shown to be directed by the transcription factor nuclear respiratory factor 1 (NRF1). In motor neurons, AUTS2 and NRF1 colocalize at actively transcribed loci, whereby AUTS2 binding requires NRF1, but NRF1 binding is independent of AUTS2 (Liu et al., 2021). Thus, it was suggested that NRF1 recruits AUTS2 in the context of the PRC1.3 complex to genes involved in neuronal differentiation. The transcription of these genes will then be activated by binding of EP300 to the AUTS2 HX repeat domain (Liu et al., 2021).

Like AUTS2, FBRSL1 was also identified by tandem affinity purification and mass spectrometry as an interaction partner of PRC1.3 and PRC1.5 (Gao et al., 2012). Further, it was shown that FBRSL1 competes with AUTS2 for binding to the PRC1.5 complex (Gao et al., 2014). Thus, it will be interesting to see if a PRC1.3 or PRC1.5 complex containing FBRSL1 in place of AUTS2 has again a repressive function instead of an active role. While interaction of FBRSL1 and AUTS2 with Polycomb complexes indicates a role of these proteins in transcriptional gene regulation, they likely also control additional processes in the cytoplasm.

For AUTS2 it has been shown that—in addition to its function in the nucleus—it also functions in the cytoplasm by controlling cytoskeletal dynamics. Cytoplasmic AUTS2 functions by regulating small GTPases of the Rho family thereby affecting actin dynamics in the developing brain (Hori et al., 2014). By stimulating small guanine exchange factors (GEFs) AUTS2 activates Rac1 and induces lamellipodia formation and neurite extension. Conversely, AUTS2 inhibits Cdc42 thereby suppressing filopodia formation (Hori et al., 2014). For Rac1 activation, the N-terminal PR1 region of the AUTS2 protein seems to be important, as overexpression of mutant AUTS2, lacking the N-terminal PR1 domain, did not lead to lamellipodia formation (Hori et al., 2014). Currently, it is unknown if FBRSL1 may play a similar role. However, we recently demonstrated that FBRSL1 is localized in the cytoplasm as well as in the nucleus of HEK293 cells and human fibroblasts (Figures 1E,F) (Ufartes et al., 2020). Consistent with the AUTS2 data (Hori et al., 2014), mainly a nuclear FBRSL1 pattern was detected with an antibody directed against the C-terminal part of FBRSL1, while nuclear and cytoplasmic FBRSL1 was observed with an antibody targeted against the N-terminal part of the protein (Figure 1F) (Ufartes et al., 2020). Thus, it is likely that FBRSL1—like AUTS2—has nuclear versus cytoplasmic functions which may require distinct domains of the protein.

## *FBRSL1* and *AUTS2* Show Transcriptional Complexity

Consistent with the concept of distinct subcellular functions, different transcripts have been identified for *AUTS2* and *FBRSL1*. The longest *AUTS2* and *FBRSL1* transcripts are encoded by 19 exons, in addition shorter N-terminal or C-terminal transcripts of the respective proteins have been described. For AUTS2, two isoforms have been extensively studied: the long transcript containing 19 exons (NM_015570.4) and a short C-terminal isoform containing the last 11 exons, starting at exon 9, first characterized by Beunders et al., 2013 (Beunders et al., 2013). The C-terminal isoform contains a region of homology to the paralogs FBRSL1 and FBRS, which was called AUTS2 family domain (Kondrychyn et al., 2017), and is critical for the nuclear function of AUTS2 (Beunders et al., 2013). Beunders et al. showed that the characteristic dysmorphic features were more pronounced in patients with 3’ AUTS2 deletions (Beunders et al., 2013). Furthermore, they showed that Morpholino-mediated knockdown of zebrafish Auts2 resulted in microcephaly and reduced lower jaw size, comparable to defects seen in patients with an *AUTS2* disruption. The morphant phenotypes could be rescued with wild-type human full-length *Auts2* RNA, but also with a short C-terminal *Auts2* isoform encoded by exons 9–19 (Beunders et al., 2013) demonstrating the important role for the AUTS2 C-terminus during development.

Like for *AUTS2*, a long *FBRSL1* transcript containing 19 exons (NM_001142641.2) was identified (Figure 1E) (Ufartes et al., 2020). In addition, two N-terminal isoforms were validated (Figure 1E) and studied in more detail. The two short isoforms contain an alternative exon three leading to a stop codon. These two short N-terminal forms lack the homologous AUTS2 family domain, but include a predicted DNA translocase domain (Ftsk) (NCBI conserved database, CDD) (Ufartes et al., 2020). Interestingly, while the severe AUTS2 syndrome phenotype was caused by variants of the C-terminus (Beunders et al., 2013), the situation was different for the three patients with the FBRSL1-associated syndromic phenotype: all three patients harbor a truncating variant (stop mutation in two patients and a frameshift variant with premature stop codon in the other patient) in the N-terminus of FBRSL1 affecting the short N-terminal isoforms (Ufartes et al., 2020). Consistently, using the *Xenopus* systems, we could show that a human N-terminal isoform of FBRSL1 was able to rescue the *Xenopus* morphant craniofacial defects. However, neither a patient variant of this isoform nor the long FBRSL1 isoform, which both lack the Ftsk domain, were able to rescue the morphant phenotype (Ufartes et al., 2020). These data suggest that mutations of the short N-terminal FBRSL1 isoforms are causative for the developmental phenotype in the animal model system and possibly also in human patients.

It is tempting to speculate that this transcriptional complexity is also responsible for the distinct functions of FBRSL1 and AUTS2. For example, in zebrafish it has been shown that the transcriptional complexity of distinct Auts2 family ohnologs is mediated by alternative splicing and alternative promotor use (Kondrychyn et al., 2017). Interestingly, the expression of the distinct Auts2 paralogs is temporally and spatially tightly controlled during development (Kondrychyn et al., 2017). Thus, there are multiple levels, by which distinct functions can be regulated by this gene family.

## Conclusion

According to currently available data on FBRSL1, we would speculate that the N-terminal region of FBRSL1, has an important function in mammalian development. This hypothesis is also supported by the finding that all three patients, affected by a novel severe malformation syndrome, carry *FBRSL1* variants localizing to the N-terminal region of *FBRSL1*. Although these patients show overlapping features to patients with AUTS2 syndrome, which is caused by variants in the *FBRSL1*-paralog *AUTS*2, they have a higher rate and wider spectrum of congenital malformations. As the number of described patients with *FBRSL1* variants are currently small, larger patient cohorts with clinical description of the disease are required to confirm these first observations. FBRSL1 and AUTS2 are closely related paralogs, but the presently published data indicate that they have distinct functions and cannot replace each other. Thus, future research will need to address the molecular and cellular mechanism of FBRSL1 to reveal its unique role in development and disease.
